# Effects of data transformation and model selection on feature importance in microbiome classification data

**DOI:** 10.1186/s40168-024-01996-6

**Published:** 2025-01-04

**Authors:** Zuzanna Karwowska, Oliver Aasmets, Estonian Biobank research team, Mait Metspalu, Andres Metspalu, Lili Milani, Tõnu Esko, Tomasz Kosciolek, Elin Org

**Affiliations:** 1 Małopolska Centre of Biotechnology, Jagiellonian University, Krakow, Poland; 2 Doctoral School of Exact and Natural Sciences, Jagiellonian University, Krakow, Poland; 3 Estonian Genome Centre, Institute of Genomics, University of Tartu, Tartu, Estonia; 4 Department of Data Science and Engineering, Silesian University of Technology, Gliwice, Poland; 5 Sano Centre for Computational Medicine, Krakow, Poland

## Abstract

**Background:**

Accurate classification of host phenotypes from microbiome data is crucial for advancing microbiome-based therapies, with machine learning offering effective solutions. However, the complexity of the gut microbiome, data sparsity, compositionality, and population-specificity present significant challenges. Microbiome data transformations can alleviate some of the aforementioned challenges, but their usage in machine learning tasks has largely been unexplored.

**Results:**

Our analysis of over 8500 samples from 24 shotgun metagenomic datasets showed that it is possible to classify healthy and diseased individuals using microbiome data with minimal dependence on the choice of algorithm or transformation. Presence-absence transformations performed comparably to abundance-based transformations, and only a small subset of predictors is necessary for accurate classification. However, while different transformations resulted in comparable classification performance, the most important features varied significantly, which highlights the need to reevaluate machine learning–based biomarker detection.

**Conclusions:**

Microbiome data transformations can significantly influence feature selection but have a limited effect on classification accuracy. Our findings suggest that while classification is robust across different transformations, the variation in feature selection necessitates caution when using machine learning for biomarker identification. This research provides valuable insights for applying machine learning to microbiome data and identifies important directions for future work.

**Supplementary Information:**

The online version contains supplementary material available at 10.1186/s40168-024-01996-6.

## Introduction

Human microbiome carries a vast amount of information that can be used to improve the understanding of our functioning and be potentially used to improve clinical practice and public health. Harnessing this information on the other hand is not trivial due to the complexity of the microbial ecosystem, which comprises hundreds of species and involves intricate interactions between the ecosystem members. Similarly to other fields of biology, machine learning (ML) approaches have become pivotal in microbiome research as they can inherently account for the high dimensionality and versatile data types. Predicting an outcome based on the taxonomic or functional profile is perhaps the most widespread use of ML in the microbiome field; however, thanks to its versatility, ML is used for taxonomic assignment, functional profiling, and others [1]. ML has been successfully used not only to build classification models for diseases such as colorectal cancer [2] and pancreatic cancer [3], but also for predicting the disease outcome in the future such as for liver diseases [4], type 2 diabetes [5], or all-cause mortality [6]. 

Currently, analysis of the microbiome data lacks standards, and the best approaches are yet to be identified [7], Hernández [8]. For example, differential abundance analysis, a common analysis step to identify members of the microbiome whose abundance is different between the study groups, has been shown to produce remarkably varying results depending on the analysis methodology used [9]. Such conflicting results can be explained by the unique properties of microbiome data, such as compositionality, high dimensionality, and high sparsity, which pose challenges for standard statistical methods and by the observation that many DA methods evaluate tests on very different estimates [10]. To address these limitations, various data transformations like total-sum-scaling (TSS), arcsine-square-root (aSIN), and log-ratio transformations such as centered-log-ratio (CLR), isometric-log-ratio (ILR), or additive log-ratio (ALR) are commonly employed in microbiome research [11]. However, the impact of data transformations on prediction and classification tasks employing machine learning algorithms remains poorly understood.

Recently, Giliberti et al. carried out an extensive analysis to compare the performance of models based on the presence-absence of microbes and TSS scaling [12]. Intriguingly, they found that presence-absence of the microbes as features in a predictive model leads to equivalent predictive performance. However, there are indications that other data transformations, especially log-ratio-based transformations can outperform the TSS in predictive tasks. For example, CLR has been shown to improve the prediction accuracy over TSS [7, 13]. Nevertheless, in light of the results by Giliberti et al., it remains unclear whether the aforementioned data transformations can improve the prediction accuracy over presence-absence.

Here, we systematically evaluate the impact of various data transformations on the binary classification performance (e.g., distinguishing healthy and diseased individuals) to determine the optimal modeling strategies for shotgun metagenomics data. We employ eight data transformations in combination with three ML algorithms (random forest, extreme gradient boosting, and elastic net) and assess their performance on 24 metagenomic datasets across various disease outcomes to ensure an unbiased and robust assessment. In addition, we investigate how the selection of the data transformation impacts the external generalizability and feature selection, which is essential for biomarker discovery.

## Results

### Study design

**Fig. 1:**
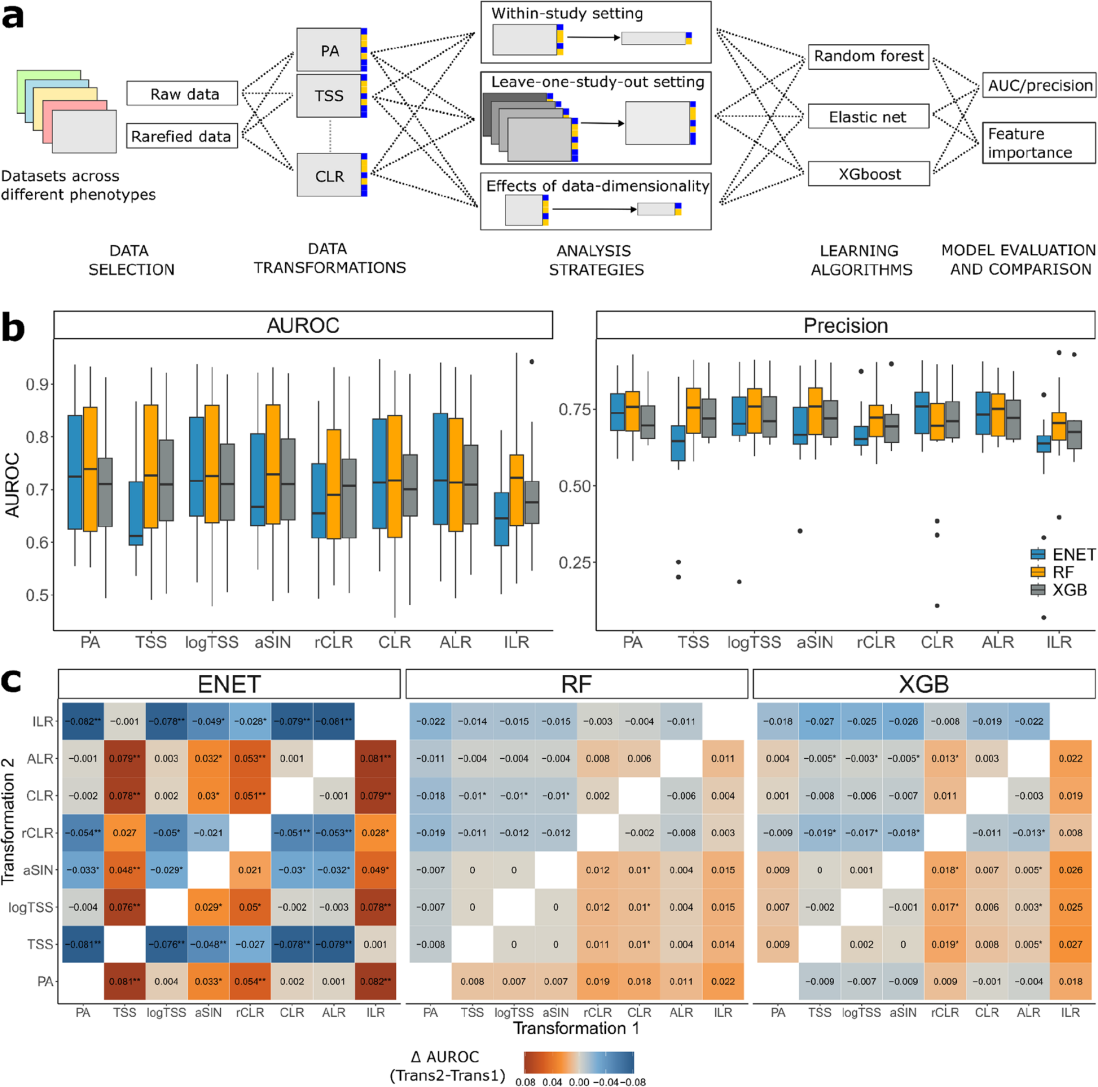
Study design and classification results.** a** Overview of the study design. **b** Classification performance (AUROC and precision) in the within-study setting for every data transformation and algorithm. **c** Statistical analysis results between the used data transformations (Wilcoxon signed-rank test) for elastic net (ENET), random forest (RF), and extreme gradient boosting (XGB). Values and colors correspond to the differences in AUROC between Transformation 2 and Transformation 1; the * symbol indicates a nominally statistically significant difference in AUROC (Wilcoxon signed-rank test, *p*-value ≤ 0.05), the ** symbol indicates a statistically significant difference in AUROC after correction (FDR ≤ 0.05). Abbreviations: ENET, elastic net logistic regression; RF, random forest; XGB, extreme gradient boosting, XGBoost; PA, presence-absence; TSS, total-sum scaling; logTSS, logarithm of TSS; aSIN, arcsine square root; CLR, centered log-ratio; rCLR, robust CLR; ALR, additive log-ratio; ILR, isometric log-ratio

To investigate the impact of the data transformations on the binary classification performance, we used publicly available shotgun metagenomic sequencing datasets present in the *curatedMetagenomicData* R package (version 3.6.2), which encompass more than 6000 samples across different populations and phenotypes [14]. In our analysis, we focused on stool metagenomic datasets with a primary phenotype available and that had at least 50 cases and 50 controls (Supplementary Table 1, Methods). Additionally, we used the metagenomic data from the Estonian Microbiome Cohort (EstMB), which is coupled with rich phenotype data (*N* = 2509) [15]. Figure 1a shows the study design and study objectives. Firstly, each metagenomic dataset was transformed using eight data transformations, which are commonly applied in the microbiome field. The transformations included presence-absence transformation (PA), relative abundance transformation (total sum scaling, TSS), logarithm of TSS, arcsine square root transformation (aSIN), and four compositional transformations (centered log-ratio (CLR), robust centered log-ratio (rCLR), isometric log-ratio (ILR), and additive log-ratio (ALR)). For sensitivity analysis, we additionally rarefied the datasets before applying data transformations. The transformed datasets were then used in a binary classification setting using three learning algorithms, random forest (RF), extreme gradient boosting (XGB), and elastic net (ENET).

Our primary objective was to assess the classifier performance across the data transformations within the analyzed datasets (within-study (WS) setting). Secondly, we aimed to assess the impact of data transformations in different analytical scenarios. In addition to the within-study setting, we evaluated the external generalizability of the models by carrying out a leave-one-study-out cross-validation for colorectal cancer (CRC; 11 datasets, Supplementary Table 1) and obesity (subjects with BMI > 30; BMI30; 5 datasets, Supplementary Table 1). Furthermore, we analyzed whether the data transformations benefit from a larger sample size and whether the corresponding model performance is dependent on the number of predictors by altering the data dimensionality and evaluating the effects on the classification performance. Lastly, we analyzed the features selected by the models to gain additional insights about how data transformations impact the conclusions of the ML analysis.

### Overview of classifier performance dependence on data transformations

Our primary aim was to analyze whether the machine learning performance in a binary classification task depends on the selected data transformation and whether the data transformations are leveraged differently by distinct ML algorithms. The comparison of the area under the receiver operating characteristic (AUROC) for random forest (RF), extreme gradient boosting (XGB), and elastic net logistic regression (ENET) by data transformations are shown in Fig. 1b, c. On average, the performance of ENET was significantly lower than RF and XGB when TSS was used as a data transformation (Wilcoxon signed-rank test, FDR ≤ 0.05). Similarly, RF outperformed ENET with ILR and rCLR and RF outperformed XGB with PA. With other data transformations, differences between the algorithm performances were not statistically significant (Fig. 1b). For ENET, XGB, and RF, rCLR performed significantly worse than several other data transformations indicating that rCLR is not fit for ML purposes. Similarly, ILR transformation led to significantly lower performances compared to other data transformations, which, however, was not statistically significant for RF and XGB. Other than that, we did not identify significant differences in classification performances between the data transformations that were universal for the learning algorithms and across different datasets (Fig. 1b, c; Supplementary Fig. 1). For ENET, ILR, rCLR, aSIN, and TSS resulted in inferior performance compared to the other data transformations (Wilcoxon signed-rank test, FDR ≤ 0.05). Importantly, PA for ENET was better or equivalent to other data transformations in terms of predictive performance. In contrast, RF and XGB did not exhibit as pronounced differences in AUROC between different data transformations, although the usage of PA with RF led to better classification performance for RF when compared to ILR, CLR, rCLR, and ALR (Fig. 1b, c). Similarly, RF in combination with TSS, logTSS, and aSIN outperformed CLR (nominal significance, *p*-value ≤ 0.05). For XGB, ALR, aSIN, TSS, and logTSS led to better performance than rCLR; other differences were not statistically significant (nominal significance, *p*-value ≤ 0.05). As a sensitivity analysis, we carried out rarefaction before applying the data transformations. In this scenario, we observed highly similar results to the non-rarefied case with PA leading to optimal predictive performance (Supplementary Figs. 2, 3). On average, the performance of the rarefied data was lower compared to the unrarefied data for aSIN (FDR = 0.0062), TSS (FDR = 0.0083), logTSS(FDR = 0.0012), and ALR (FDR = 0.0155) indicating that for binary classification on the shotgun metagenomics data, rarefaction is not necessary. Thus, our results are consistent with the results by Giliberti et al. ((2022) showing that presence-absence (PA) leads to equivalent or even better classification performance as compared to using relative abundances. Moreover, our results show that the same can be concluded for other commonly used data transformations.

### Data transformation effects in different analytical scenarios

**Fig. 2:**
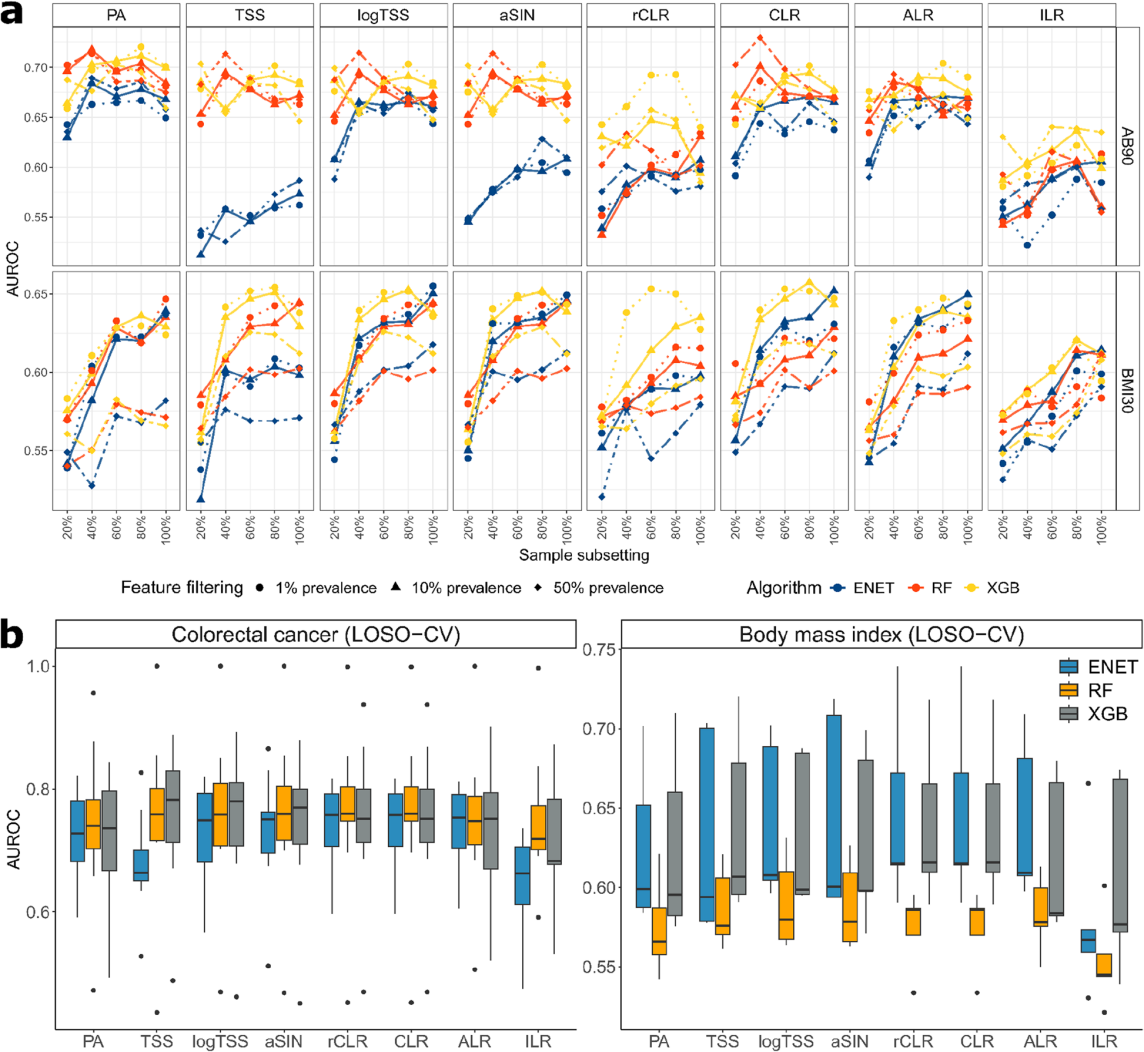
Impact of data transformations on classification performance under different scenarios.** a** Classification performance across varied data dimensions and sample sizes for random forest (RF), extreme gradient boosting, XGBoost (XGB), and elastic net logistic regression (ENET) and every transformation. **b** Transformation and model outcomes in a leave-one-study-out cross-validation (LOSO-CV) setting. Abbreviations: ENET, elastic net logistic regression; RF, random forest; XGB, extreme gradient boosting, XGBoost; PA, presence-absence; TSS, total-sum scaling; logTSS, logarithm of TSS; aSIN, arcsine square root; CLR, centered log-ratio; rCLR, robust CLR; ALR, additive log-ratio; ILR, isometric log-ratio; AB90, antibiotics use within 90 days from sample collection; BMI30, obesity as defined by BMI > 30

We were surprised that no significant improvement in classification performance was observed when abundance-based transformations were used instead of PA. To understand whether the data transformations could give advantage in other analytical scenarios, we conducted several follow-up analyses. Firstly, we assessed how the sample size and number of features in the initial dataset influenced the classification performance. We hypothesized that some data transformations may lead to better performance in certain sample size/data dimensionality settings. For that, we applied different prevalence thresholds to the microbial taxa on the publicly available metagenomics datasets and on the Estonian Microbiome Cohort (EstMB) dataset (*N* = 2509) before carrying out the classification task. For the EstMB dataset, we additionally subsampled the cases and controls of obesity (BMI > 30) and antibiotic usage (90 days from sample collection) (20%, 40%, 60%, and 80% of the initial number of cases and controls) to study the impact of varying sample size and number of predictors at once. As expected, we observed that larger sample sizes and the inclusion of less prevalent taxa lead to improved classification performance (Fig. 2a, Supplementary Fig. 2). Nevertheless, we found no substantial interactions on the classification performance between the data transformations, sample size, and the number of features.

Next, we assessed whether data transformations impacted the model’s ability to generalize to unseen data by measuring its classification performance on an external dataset. To do this, we employed a leave-one-study-out (LOSO) validation method for both colorectal cancer (CRC) and obesity defined by BMI > 30 (BMI30) datasets. This involved training a model on a combined set of samples from all studies except one and evaluating its performance on the omitted study. Similarly to the within-sample setting, we observed no significant improvement in the model generalizability when employing abundance-based data transformations (Fig. 2b). Thus, our analysis indicates that in terms of classification performance, presence-absence is usually a good option and should be considered an alternative to the abundance-based transformations.

### Feature importance

**Fig. 3:**
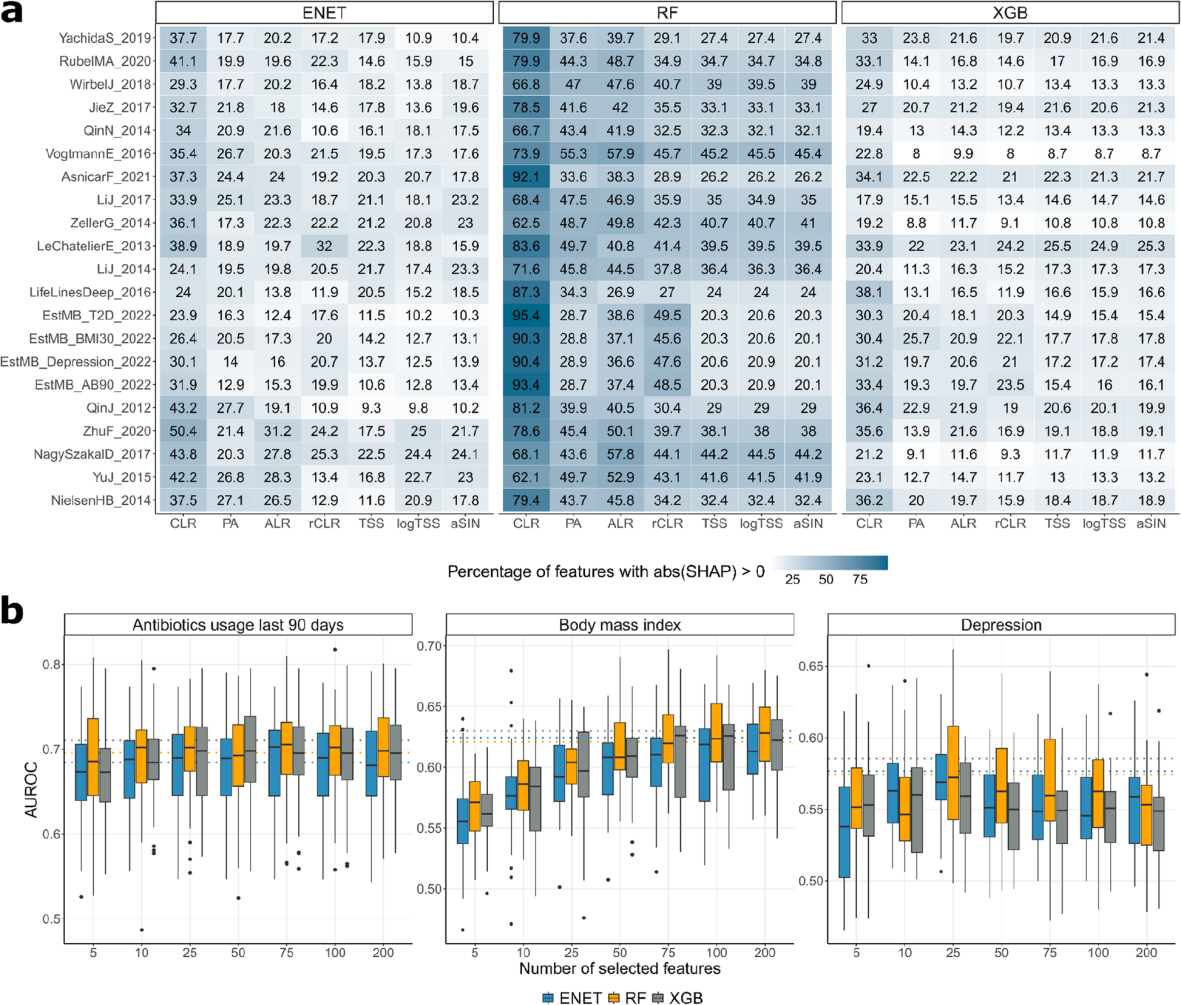
Feature selection among data transformations.** a** Number of selected features: proportion for ENET, XGB, and RF. **b** XGB, ENET, and RF classification performance on presence-absence (PA) data using top-N features. The dashed line indicates classifier performance on all features

As no data transformation could consistently be considered superior in terms of classification performance and several data transformations led to equivalent performance, we were interested in how different data transformations impact feature selection and feature importance. To assess feature importance, we calculated mean absolute SHapley Additive exPlanations (SHAP) values for each dataset and for each microbe. SHAP values are a method used in machine learning to explain the contribution of each feature to the prediction of a model [16]. Focusing on predictors with a non-zero mean absolute SHAP value, we found that the number of selected predictors was highly transformation-specific. Compositional transformations ALR, CLR, and rCLR selected more predictors, particularly when used with RF (Fig. 3a). However, regardless of the transformation, only a small subset of features (~ below 25 features) held significant importance (Supplementary Figs. 4, 5). To validate this, we built classifiers for obesity, depression, and antibiotics use on the PA-transformed EstMB cohort data that cumulatively use only the most significant features. Surprisingly, just 10 most significant microbial predictors for antibiotics, 25 for depression, and 75 for BMI resulted in comparable classification performance when compared to models using the full microbiome profile, with performance decreasing as more features were added (Fig. 3b, Methods). We believe the decline in model performance with additional features is due to the unique characteristics of gut microbiome data. Its compositional, high-dimensional nature can cause overfitting, especially with small sample sizes. Using fewer key features helps reduce noise and improves model accuracy. Dimensionality reduction methods such as principal component analysis (PCA), non-metric multidimensional scaling (NMDS), or non-negative matrix factorization (NMF) can address these challenges by transforming highly correlated features into orthogonal vectors. However, these techniques come with limitations, including the need for careful data transformation, appropriate pseudocount use, and accounting for phylogenetic interactions. Additionally, understanding which features drive classification becomes harder to interpret with these methods [17].

**Fig. 4:**
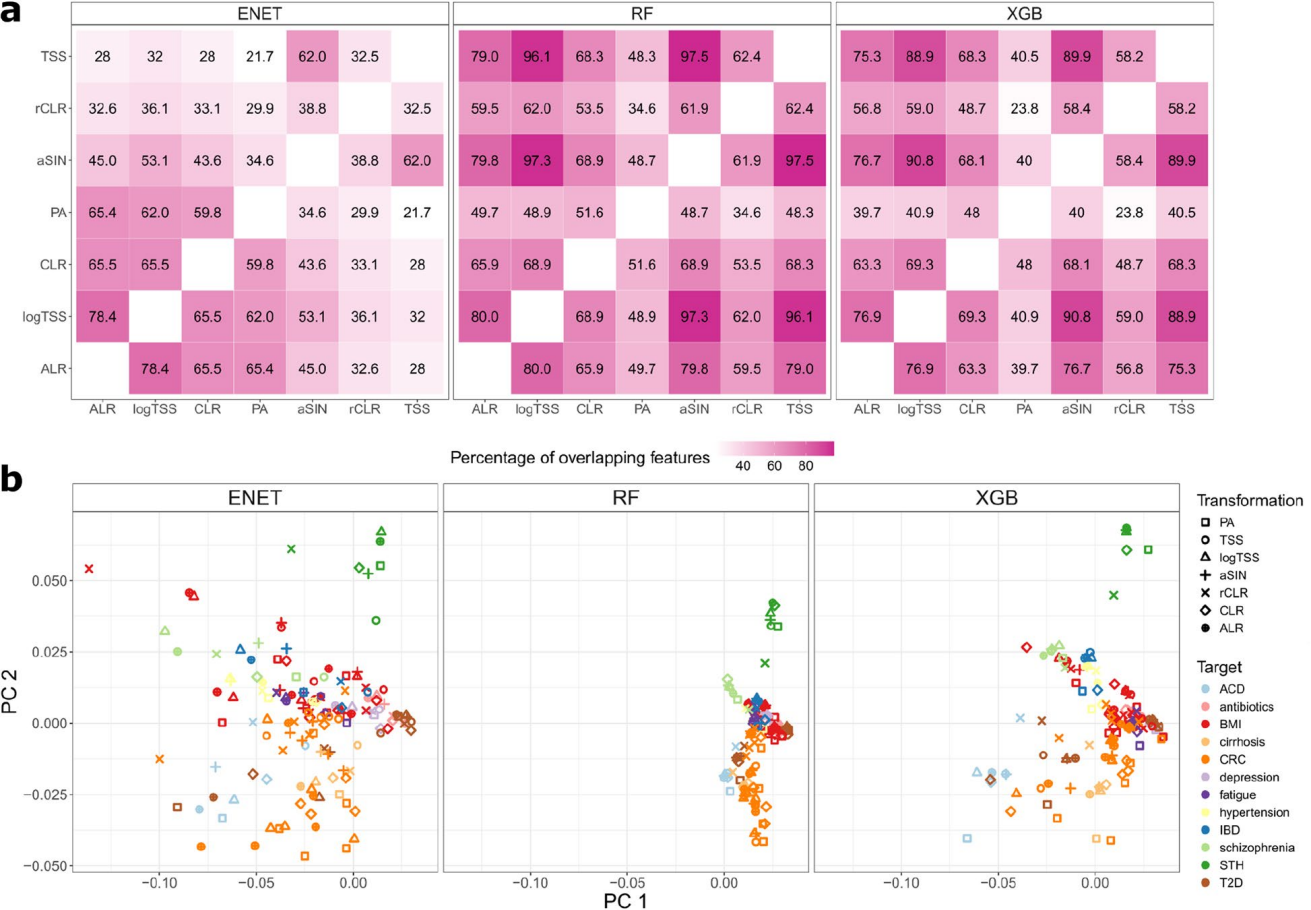
Stability of feature importance results. **a** Overlap of top-25 features between transformations for each algorithm and dataset. **b** Principal component analysis of the SHAP profiles visualized separately for ENET, XGB, and RF

Next, we examined the features with the highest predictive value across the transformation-algorithm combinations. Building on our earlier results highlighting a small subset of features with high SHAP values, we first focused on the overlap among the top 25 predictors exhibiting the highest mean absolute SHAP values (Fig. 4a). For ENET, TSS, rCLR and to lesser extent aSIN exhibited lower overlap with other transformations, while the highest agreement was found between PA, CLR, ALR, and logTSS. The overlap between the top predictors for RF was remarkably higher with TSS, aSIN, and logTSS showing almost perfect correspondence. The top predictors for XGB closely aligned with those from RF, showing strong similarity across TSS, aSIN, and logTSS transformations. Interestingly, although the classification performance of PA with RF was comparable or superior to CLR, rCLR, ALR, and logTSS, the overlap among the most significant predictors is lowest for PA with around 50% correspondence to the predictors informed by the abundance-based data transformations. Thus, different abundance-based transformations can inform highly similar biomarker candidates, but the selection might not be optimal for classification performance indicating that PA transformation is able to indicate potentially novel biomarkers of equal predictive value. Following the observation of poor prediction performance of ENET in combination with TSS and rCLR, the informed features are also highly distinct from predictors informed by the other data transformations. Thus, our results raise caution against biomarker detection using ENET in combination with TSS. When comparing the predictors informed by RF and ENET, around 50% of the predictors overlap across the transformations. Here, PA is a notable exception with significantly higher overlap between the features informed by RF and ENET. Interestingly, RF and XGB showed a high percentage of overlapping features regardless of the data transformation used, with PA (presence-absence) having the lowest overlap. This lower overlap might stem from the fact that other transformations tend to identify a larger number of important features compared to the presence-absence data transformation (Supplementary Fig. 6).

Next, we assessed how similar in general are the feature importance profiles informed by different data transformations and algorithms. For that, we calculated Euclidean distances on the SHAP value profiles and carried out principal component analysis (Fig. 4b). The results show that the target-specific signatures are clearly evident independent of the learning algorithm and data transformation. For example, predictive signatures for colorectal cancer (orange) and soil-transmitted helminths (green) clearly stand out along the PC2 axis. However, there is still a remarkable difference between the algorithms with RF displaying less variation in the feature importance profiles compared to ENET and XGB. Although feature importance profiles resulting from different data transformations are more similar within the same study than they are between studies indicating a detected target-specific signal, the effect is stronger for RF (Supplementary Fig. 7). Thus, RF identifies a more target-specific signal and is less affected by the choice of the data transformation. Interestingly, the feature importance profiles for ENET and XGB are more similar across studies when the same data transformation is used than they are across studies and across different data transformations (*p* = 1.4e-05 for ENET and *p* = 0.0092 for XGB). Thus, ENET and XGB can identify signatures which are not only specific to the target variable but are common across the datasets. The same is not true for RF (*p* = 0.17).

Given the differences in the most informative predictors, particularly between PA and abundance-based transformations, we were interested in whether the feature selection is associated with the prevalence and relative abundance of the microbial taxa. For that, we calculated Pearson correlations between SHAP values and taxa prevalence and relative abundance (Supplementary Fig. 8). Expectedly, we found that the SHAP values are positively correlated with the taxa prevalence. However, the correlation with prevalence was lower for the features informed by PA and CLR independent of the learning algorithm. Similarly, we observed that the SHAP values obtained by abundance-based transformations exhibit remarkably higher correlations with the relative abundance as compared to SHAP values obtained by PA. This can partially be the reason why ENET and XGB choose similar predictors across different target variables. To further understand how this correlation is reflected in the feature selection, we analyzed the microbial predictors, which had the largest difference in SHAP values between TSS and PA (Supplementary Figs. 9, 10, 11). For all algorithms, more abundant taxa have higher significance with TSS when compared to PA. Moreover, the bacteria which were more important with the TSS include well-known and abundant gut commensals such as *Prevotella copri* [18], *Bacteroides vulgatus* [19],* Bacteroides uniformis* [20], and *Faecalibacterium prausnitzii* [21]. Bacteria having the highest SHAP values when using any model with PA were probiotic candidates such as *Akkermansia municiphila* [22] and potentially beneficial microbes such as *Firmicutes bacterium CAG:95* and *Firmicutes bacterium CAG:110* [23]. Models using PA as data transformation assigned also higher SHAP values to opportunistic pathobionts such as *Desulfovibrio piger* [24], *Fusobacterium nucleatum* [25], and *Erysipelatoclostridium ramosum* (previously known as *Clostridium ramosum*) [26]. This observation further highlights the difference in the biomarker profiles informed by different data transformations. With abundance-based transformations, we are more likely to identify more prevalent and abundant taxa which might potentially complicate the identification of disease-specific markers. In contrast, the biomarkers informed by PA are largely independent of these properties.

In conclusion, our findings underscore that models assign high importance to a limited set of microbes, a property common for all data transformations. However, despite obtaining similar classification performance, there can be large differences in the most informative features chosen by the transformations. This variability could impact the development of gut microbiome health indices, assessment of microbiome dysbiosis, and biomarker discovery. Together with the equivalency in the resulting classification performance, this highlights the need to improve the feature identification, validation, and stability.

## Discussion

Our goal was to evaluate the impact of data transformations on machine learning performance in microbiome binary classification [1]. We compared the classification performance of two learning algorithms in combination with seven data transformations across various analytical scenarios and analyzed the impact of using different data transformations on the feature importance. Our results showed that there was no significant improvement in classification performance when abundance-based transformations were used instead of presence-absence (PA). This result is consistent with the comparison of PA and TSS as reported by Giliberti et al. [12], but we further extend their findings to several other commonly used data transformations. Similarly to Giliberti et al., we observed that an elastic net algorithm (ENET) performs better with PA when compared to total-sum scaling (TSS), but there is no major difference when random forest (RF) or XGBoost (XGB) are used. Interestingly, all three models showed a decline in performance when applied with the isometric log-ratio (ILR) transformation, with the most significant drop observed in ENET. Although we saw the previously reported improved performance of ENET in combination with CLR as compared to TSS [27], our results did not confirm the benefit of using abundance-based data transformations as reported in several other studies. For example, in combination with RF, centered log-ratio (CLR) has been shown to outperform TSS and logTSS [7]. Moreover, based on our results, there was no significant effect of the transformation selection on the model generalizability nor interaction with the model performance and data dimensionality. The performance of machine learning models seems to be more reliant on the characteristics of the dataset than on the specific algorithm or transformation employed. This principle echoes the “no free lunch theorem” in machine learning, emphasizing that no single algorithm universally outperforms others across diverse datasets [28].

Our feature analysis showed that the number of significant predictors informed by the model was more tied to the dataset than the transformations used. Importantly, there was only partial overlap among the top features informed by different data transformations, indicating that different sets of microbial features can yield similar classification performance. This observation was most pronounced for PA in combination with RF which had the lowest overlap with the other data transformations, although PA led to better predictive performance. Thus, microbiome studies could take advantage of the methodologies such as statistically equivalent signatures, which aim to identify variable sets with equal predictive power [29]. Analyzing the properties of the selected features shows that abundance-based transformations may focus on the most abundant gut microbes such as *P. copri*, *F. prausnitzii*, *B. vulgatus*, and *B. uniformis*. As using relative abundances as a data representation is one of the most popular choices for applying ML on the microbiome data [1], we suggest taking caution when focusing on the feature importance as the selection might not yield a signal specific to the condition of interest and it could potentially obscure the interpretation of classification outcomes. These findings underscore the need for further research.

Based on our findings, we recommend adopting the presence-absence (PA) transformation for microbiome data classification tasks as a supportive alternative to the abundance-based transformations. PA-based classifiers demonstrate strong performance and offer a simpler interpretation, avoiding the need for pseudocount imputation or data scale transformation. However, selecting a threshold for microbe absence (e.g., setting it at zero) introduces challenges related to structural zeros and sequencing depth, which warrants further investigation [30].

Our study’s strengths lie in its systematic approach, employing two learning algorithms on diverse datasets, which enhances generalizability. However, we acknowledge limitations. Our goal was to focus on commonly used data transformations in microbiome research to provide a solid foundation for researchers applying machine learning to binary classification tasks. While more advanced transformations like PhyILR [31] and Phylofactorization [32] can offer enhanced performance, they often rely on specific biological priors, such as balances or phylogenetic data. For this study, we prioritized general transformations that do not require specialized biological inputs, making them more broadly applicable across a variety of datasets and research contexts. Our focus is on classification tasks, potentially limiting relevance to other analyses. Dataset constraints and unexplored confounding variables are also noted. To further understand the effect of data transformation on classification accuracy and feature selection [33], taking advantage of synthetic microbial communities where alterations in bacteria are documented and ground truth is available would be beneficial. Such an approach is being used for example in silico gut microbiome community design [34–36].

In the future, we plan to explore additional questions. Firstly, we would like to assess whether using only presence-absence (PA) data is sufficient for accurate classification or if combining PA with variations in the abundance of key bacteria enhances performance. We would also investigate if the observed similarity between PA-based classifiers and abundance-based transformations is influenced by technical factors, such as shallow sequencing, which might mask certain features. Additionally, we aim to gather more datasets to assess whether SHAP values for bacterial features in each disease remain consistent across geographic locations. Moreover, we would like to extend our study and evaluate how data transformations impact various types of data modeling tasks, including regression models and unsupervised techniques like clustering. These inquiries highlight the complexity of our findings and provide directions for further research.

## Methods

### Data acquisition

#### Open data

Datasets available in the *curatedMetagenomicData* R package were used for the analysis (version 3.6.2) [14]. The *curatedMetagenomicData* package contains uniformly processed shotgun metagenomic sequencing human microbiome data of healthy and diseased subjects. The microbiome data preprocessing, including taxonomic profiling, is carried out using the bioBakery 3 toolki [37]. For the within-study (WS) evaluation setting, we focused on 17 distinct human stool metagenomics datasets, which had a primary endpoint available and included at least 50 cases and 50 controls to allow proper model evaluation. Also, we included four datasets where we defined obesity as a binary outcome defined as BMI > 30. In some instances, filtering was applied to the original dataset to achieve a binary classification task. For example, we excluded adenomas from the Zeller et al. study and focused only on colorectal cancer cases and controls [38]. The selected datasets, sample size, defined binary classification task, and the filtering procedures are shown in Supplementary Table 1.

For the leave-one-study-out (LOSO) evaluation setting, we included additional 6 colorectal cancer datasets with less than 50 cases or controls and 4 datasets, where primary endpoint was not available.

### Estonian microbiome cohort

The Estonian Microbiome cohort (EstMB) is a volunteer-based cohort currently including 2509 subjects, who have provided blood, oral, and stool samples. Being part of a larger nation-wide Estonian Biobank (EstBB), linkings to various electronic health records (EHR) and questionnaires covering the lifestyle and dietary preferences are available for all of the subjects. The cohort is described in detail in Aasmets et al. [15]. For the binary classification, four target variables were considered: antibiotics usage within the previous 90 days before the microbiome sample collection (AB90), obesity defined as BMI > 30 (BMI30), type 2 diabetes (T2D), and depression (Supplementary Table 1).

Taxonomic profiling on EstMB was carried out using *Metaphlan3* [37] to comply with the profiling done for the *curatedMetagenomicData* R package datasets.

### Data transformations

Numerous data transformations have been proposed to be used in the analysis of microbiome data. In the current manuscript, the following data transformations were considered:

1. Relative abundance/total-sum-scaling (TSS): The standard and most widely used technique, which scales data to relative abundances.
2. Log(TSS): A logarithmic transformation applied to TSS-normalized data.
3. Presence-absence (PA): changes abundance data into binary data. We used zero as the threshold for presence-absence.
4. Arcsin square root (aSIN [13]): Which involves applying the arcsin (inverse sine) function to each value, which can be useful for normalizing and stabilizing data that represents proportions or percentages, particularly in statistical analyses of compositional data.
5. Centered log-ratio transformation (CLR [39]): procedure that enhances compositional data for standard statistical analysis by dividing each value by the geometric mean of all features and applying a logarithmic transformation.
6. Robust CLR transformation (rCLR [40]): a robust version of CLR that handles zeros by using only observed taxa for the geometric mean calculations.
7. Additive log-ratio transformation (ALR [39]): in which each feature in a dataset is divided by a selected reference feature and then logarithmically transformed. We randomly selected 5 features as reference elements and averaged all the results over the different ALR transformations to account for the variability arising from the reference element selection. We observed that averaging over 5 different reference elements resulted in a reasonable trade-off between computational burden and variability of the performance estimates (Supplementary Fig. 12).
8. Isometric log-ratio transformation (ILR (Egozcueet al. 2003)): in which the compositional dataset is transformed by representing each feature as a set of orthogonal log-ratios using a basis that maintains the geometric structure of the data. The ILR transformation was applied using the implementation in the R package *compositions*.

### Machine learning pipeline

Each of the considered transformations was applied to a dataset, and a binary classification task was carried out. Random forest (RF), XGBoost (XGB), and elastic net (ENET) penalized logistic regression were used as the learning algorithms. ENET logistic regression is a machine learning algorithm that combines L1 (Lasso) and L2 (Ridge) regularization techniques to perform logistic regression with variable selection, making it suitable for high-dimensional data by minimizing overfitting and selecting the most relevant features. Regularization helps prevent overfitting by adding a penalty to the model’s complexity. L1 regularization (Lasso) encourages sparsity by shrinking some weights to zero, which helps feature selection. L2 regularization (Ridge) distributes penalties evenly across all weights, reducing their magnitude but keeping all features. A balance between L1 and L2 (Elastic Net) combines these benefits, offering both feature selection and weight regularization, helping the model generalize better [41]. Random Forest, on the other hand, is an ensemble learning method that builds multiple decision trees and combines their predictions to improve classification accuracy and handle complex relationships in data while reducing the risk of overfitting [42]. XGBoost is an advanced ensemble learning algorithm that builds multiple decision trees sequentially, where each tree corrects the errors of the previous ones, thereby improving the overall prediction accuracy. It is designed to be highly efficient and scalable, handling large datasets with complex relationships while preventing overfitting through regularization [43]. For every algorithm, we performed hyperparameter tuning using cross validation in combination with grid search, and the model with optimal hyperparameters was then used for classification. For ENET, both penalty and mixture parameters were tuned; for RF, the number of predictors to sample at each split (*mtry*) and the number of observations needed to keep splitting nodes (*min_n*) were tuned; the number of trees was 500; for XGB, the number of predictors to sample at each split (*mtry*) and the number of observations needed to keep splitting nodes (*min_n*), the maximum depth of each tree (*tree_depth*), learning rate, and the fraction of training data used for growing each tree (*sample_size*) were tuned; the number of trees was 500. 

These algorithms were chosen due to their popularity and competitive performance in the microbiome field [44], and as RF being a nonlinear, XGB being non-linear and using boosting, where trees are built sequentially, with each tree focusing on correcting the mistakes of the previous ones and ENET a linear method, they can provide insights on the impact of algorithm selection in microbiome studies. Followingly, the model fitting and evaluation are described.

### Within the study (WS) setting

For each 21 datasets used for the within-study evaluation, the following repeated holdout validation procedure for parameter tuning and model evaluation was carried out:

1. *Data is split to training/test set (75–25%) stratified by the target variable*
2. *Hyperparameter tuning on the training set (75%) using fivefold cross-validation with grid search (10 parameter combinations)*
3. *Model with optimal hyperparameters is fit on the whole training data (75%)*
4. *Model is evaluated on the test set (25%)*

The initial data test/train split and model evaluation were carried out on 10 random data splits to assess the variation arising from sampling resulting in 10 performance estimates per evaluation.

### Leave-one-study-out (LOSO) setting

The LOSO setting was carried out for the 11 available colorectal cancer and 5 obesity (BMI > = 30) datasets. The aim was to understand whether the dataset-to-dataset generalization performance might be dependent on the chosen data transformation. For the model fitting and evaluation, the following procedure was carried out:

1. *Data is split to training/test set so that one dataset works as the test set and other datasets as a combined training set*
2. *Hyperparameter tuning on the training set using fivefold cross-validation with grid search (10 parameter combinations)*
3. *Model with optimal hyperparameters is fit on the whole training data (75%)*
4. *Model is evaluated on the test set—left out dataset*

The model evaluation was carried out using each dataset per target variable as a test set. This resulted in 11 performance estimates for colorectal cancer and 5 for obesity.

### Cumulative classifier

This analysis aimed to evaluate whether a subset of the most significant predictors can be used to build a model that has optimal prediction performance. This experiment was based on the PA-transformed EstMB datasets and carried out for antibiotics usage, obesity (BMI30), and depression. For the model fitting, feature selection, and evaluation, the following procedure was carried out:

### Stage 1

1. *Data is split to training/test set (50–50%) stratified by the target variable*
2. *Hyperparameter are tuned (fivefold cross-validation with grid search) and feature importance calculated on the training set (50%)*
3. *Subsets of most important features are created (set sizes 5, 10, 25, 50, 75, 100, 200)*

### Stage 2

4. *Test data from stage 1 (50%) is used for model evaluation*
5. *For each subset of features:*
  - *Stage 1 test data is split to stage 2 training/test set (75–25%) stratified by the target variable*
  - *Hyperparameter tuning on the stage 2 training set (75%) using fivefold cross-validation with grid search (10 parameter combinations)*
  - *Model with optimal hyperparameters is fit on the whole stage 2 training data (75%)*
  - *Model is evaluated on the stage 2 test set (25%)*

The stage 1 and stage 2 data test/train splits and model evaluation were carried out on 10 random data splits to assess the variation arising from sampling.

### Model comparison

To test the differences in the binary classification results, we used the Wilcoxon signed-rank test for each pair of data transformations. For that, we first averaged the results over different folds and target variables to account for the overrepresentation of certain phenotypes like colorectal cancer and obesity. After that, a paired test within each target variable was carried out. Thus, we tested the hypothesis that the difference in the performance of *transformation1* and *transformation2* is not equal to 0 (Fig. 1c). To account for the multiple testing, the Benjamini-Hochbergi procedure was applied to the nominal *p*-values.

### Filtering subjects and features

Due to the large sample size, EstMB dataset was further used to study the effects of sample size and number of bacteria used by the model on the performance of the classifiers, focusing on differences between the data transformations. For that reason, the cases and controls of antibiotics usage (AB90) and obesity (BMI30) were subsampled to 20/40/60/80% of the initial number of cases and controls. Additionally, in combination with the sample subsetting, prevalence filtering (1/10/50%) for the microbial taxa was applied to study the impact of the number of predictors. Thus, in total of 5 × 3 = 15 scenarios per algorithm and data transformation were analyzed, and the same machine learning procedure as described in the within-sample setting evaluation was carried out.

Prevalence filtering (10/25/50/75/90%) was also carried out on the *curatedMetagenomicData* package datasets before applying the machine learning procedure as described in the within-sample setting evaluation was carried out.

### Feature importance analysis

Feature importance evaluation was based on the SHapley Additive exPlanations (SHAP) values. SHAP values are a method used in machine learning to explain the contribution of each feature to the prediction of a model [16]. We first determined the percentage of features within each dataset that exhibited mean absolute SHAP values exceeding zero. For feature overlap assessment, we quantified the average percentage of overlapping features among the top 25 features between data transformations and algorithms. The average feature overlap was calculated by taking the average across different folds and datasets. Pearson correlation was used to study the associations between SHAP values, bacterial relative abundances, and prevalence. The overall similarity of the feature importance profiles was evaluated by comparing the Euclidean distances between the feature importance profiles across the algorithm-transformation pairs. A standard *t*-test was used to formally test the differences between groups of interest (Supplementary Fig. 7). Principal component analysis on the feature importance profiles was carried out to visualize the differences between different data transformations and datasets in a two-dimensional space.

To identify microbial predictors with the largest difference in SHAP values between TSS and PA, we first calculated the mean absolute SHAP value for each feature, study, data transformation, and algorithm. Then, for each feature, study, and algorithm, we calculated the delta between the mean SHAP values for TSS and PA and averaged the delta overall studies. The top 100 features according to the absolute delta for algorithms separately were then visualized (Supplementary Figs. 9, 10, 11).

## Supplementary Information

 Additional file 1: Figs. S1–S12.

Additional file 2: Table S1.

## Data Availability

The metagenomic data generated in this study have been deposited in the European Genome-Phenome Archive database (https://www.ebi.ac.uk/ega/) under accession code EGAS00001008448. The phenotype data contain sensitive information from healthcare registers. They are available under restricted access through the Estonian biobank upon submission of a research plan and signing a data transfer agreement. All data access to the Estonian Biobank must follow the informed consent regulations of the Estonian Committee on Bioethics and Human Research, which are clearly described in the Data Access section at https://genomics.ut.ee/en/content/estonian-biobank. A preliminary request for raw metagenome and phenotype data must first be submitted via the email address releases@ut.ee.
