# Supplementary material for: Effects of data transformation and model selection on feature importance in microbiome classification data
Source: Microbiome. 2025 Jan 4;13:2. doi: 10.1186/s40168-024-01996-6 (PMC11699698; doi:10.1186/s40168-024-01996-6)
Supplement: Supplementary file 1 — Additional file 1: Figs. S1–S12. [file 40168_2024_1996_MOESM1_ESM.docx]

# Supplementary Figures


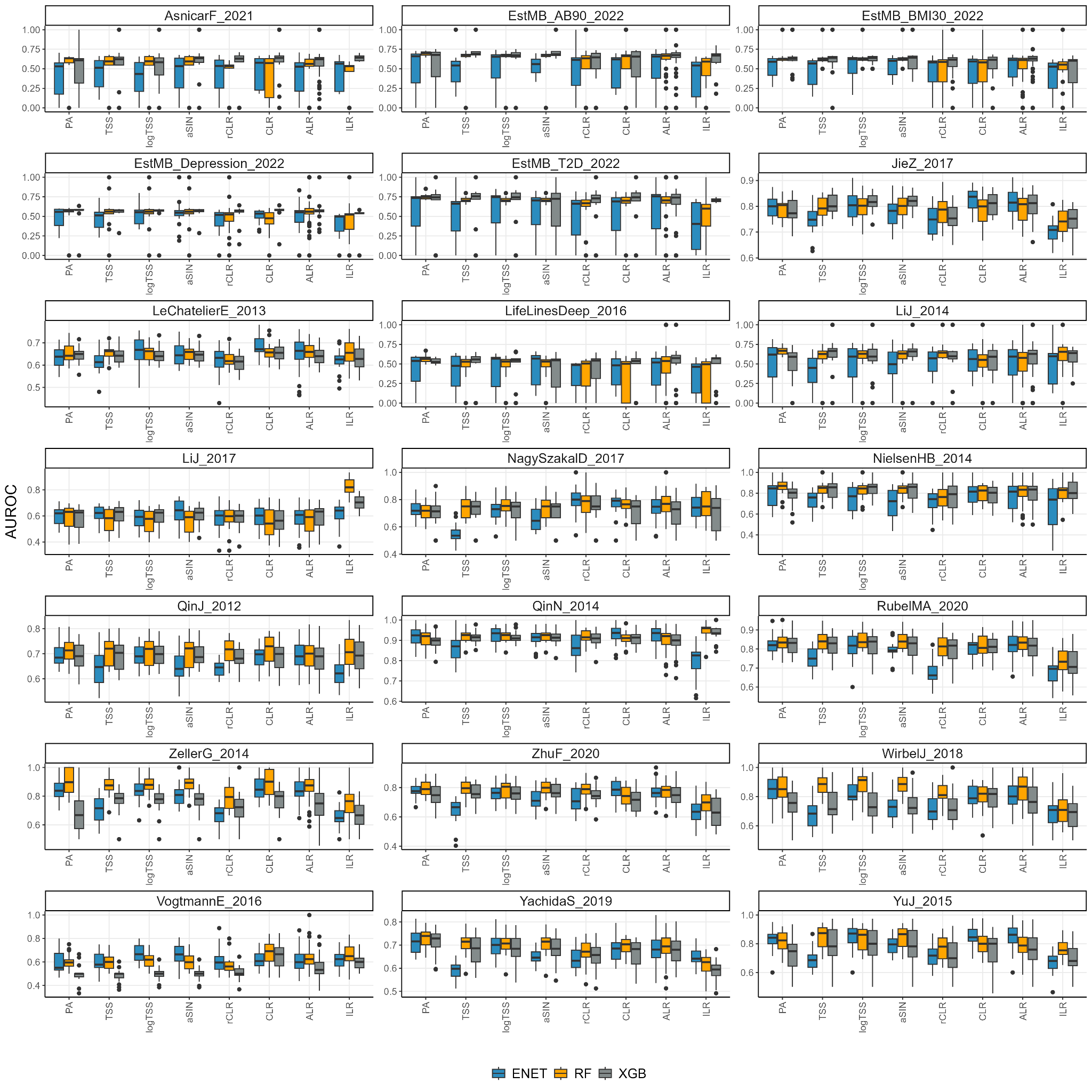


**Supplementary Figure 1**. Classification performance (AUROC) by studies. Abbreviations: *ACD - atherosclerotic cardiovascular disease;* BMI30 *- body mass index > 30; CRC - colorectal cancer; IBD - inflammatory bowel disease; STH - soil-transmitted helminths*; *T2D - type 2 diabetes*


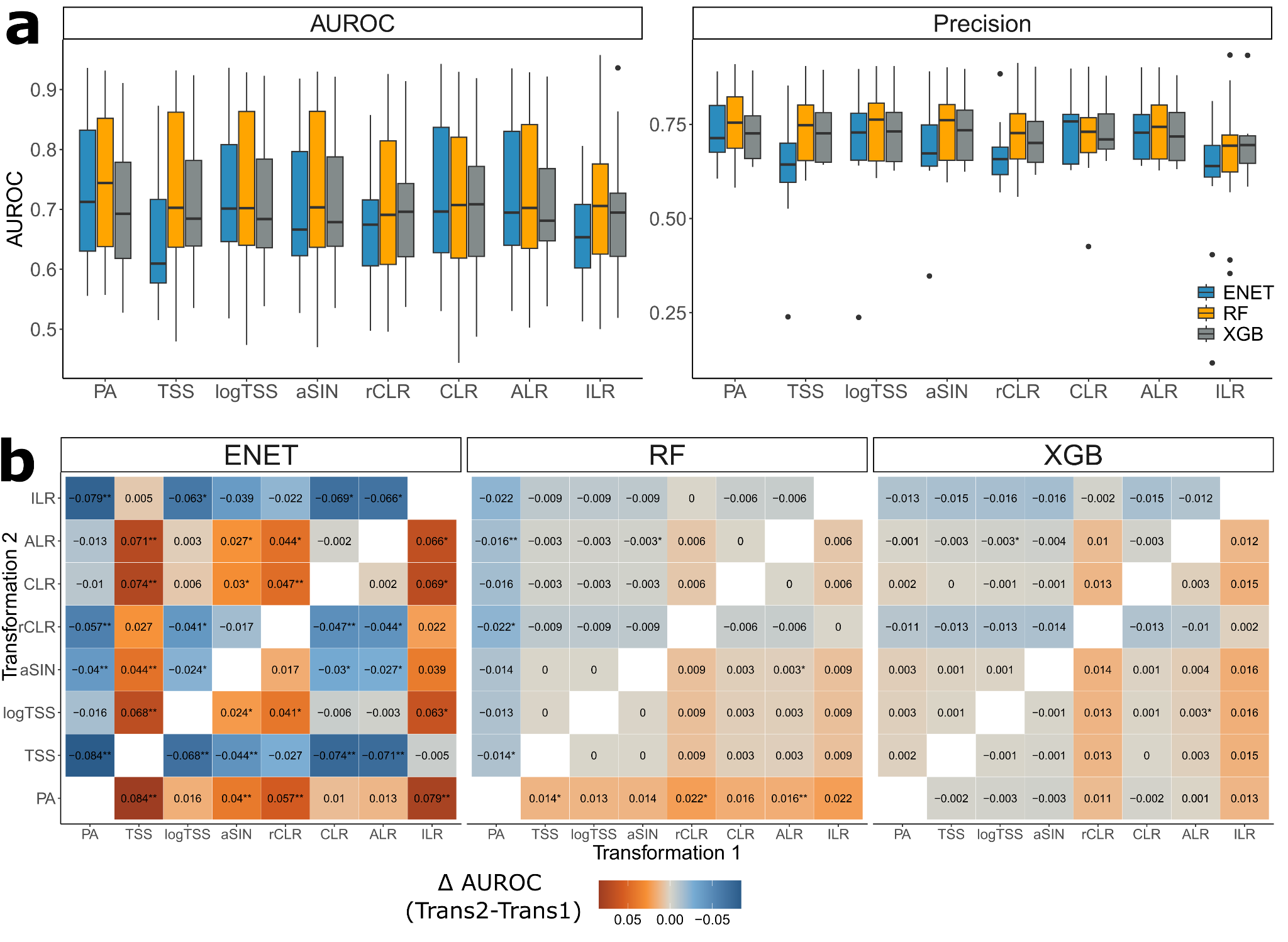


**Supplementary Figure 2. a** - Classification performance (AUROC and precision) in the within-study setting for every data transformation and algorithm for the rarefied data. **b -** Statistical analysis results between the used data transformations for rarefied data (Wilcoxon signed-rank paired test) for elastic net (ENET), random forest (RF) and extreme gradient boosting (XGB); Values and colors correspond to the differences in AUROC between Transformation 2 and Transformation 1; * indicates a nominally statistically significant difference in AUROC (Wilcoxon signed-rank test, p-value ≤ 0.05), ** indicates a statistically significant difference in AUROC after correction (Wilcoxon signed-rank test, FDR ≤ 0.05). Abbreviations: ENET - elastic net logistic regression; RF - random forest; XGB - extreme gradient boosting, XGBoost; PA - presence-absence; TSS - total-sum scaling; logTSS - logarithm of TSS; aSIN - arcsine square root; CLR - centered log-ratio; rCLR - robust CLR; ALR - additive log-ratio; ILR - isometric log-ratio.


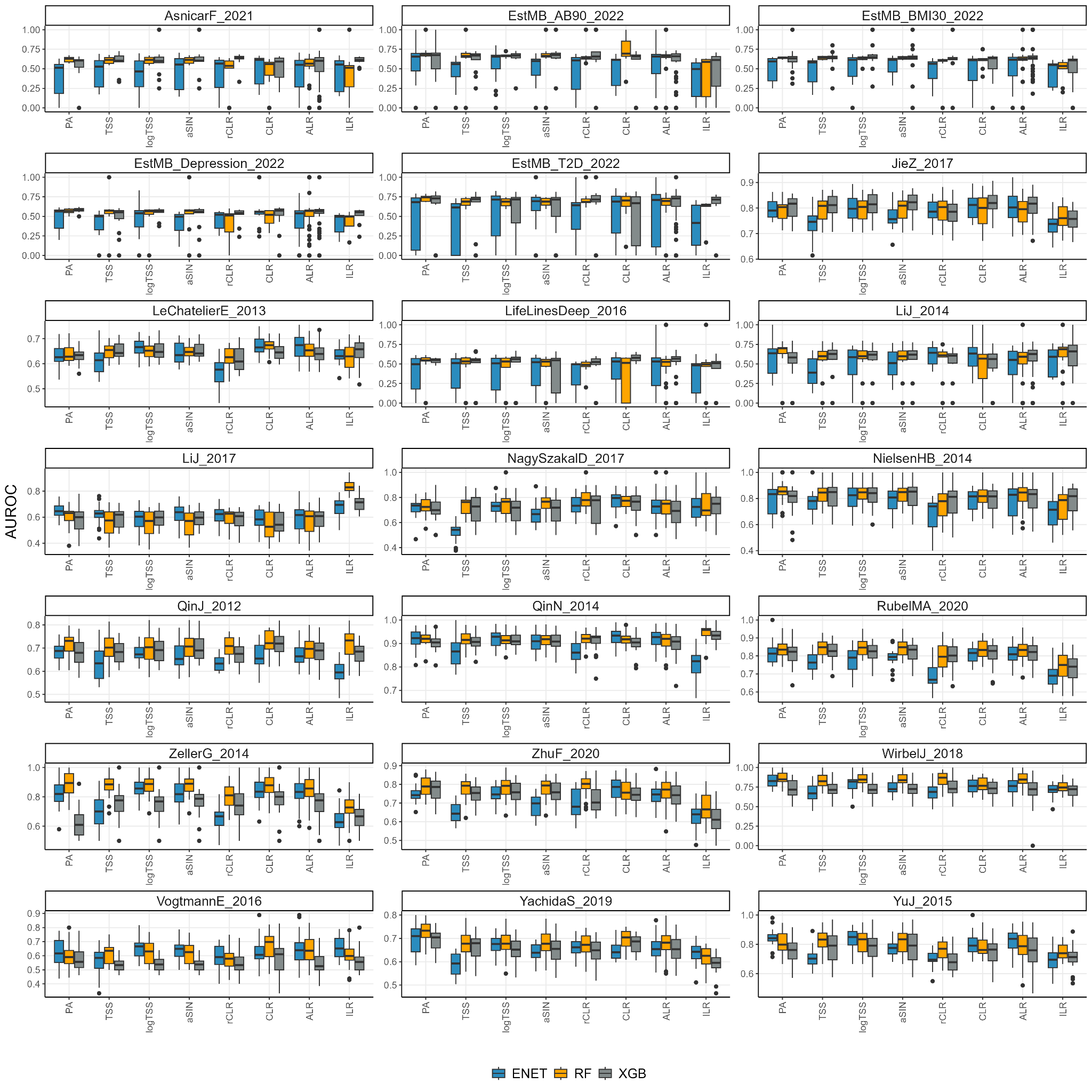


**Supplementary Figure 3**. Classification performance (AUROC) by studies on rarefied data. Abbreviations: *ACD - atherosclerotic cardiovascular disease;* BMI30 *- body mass index > 30; CRC - colorectal cancer; IBD - inflammatory bowel disease; STH - soil-transmitted helminths*; *T2D - type 2 diabetes*


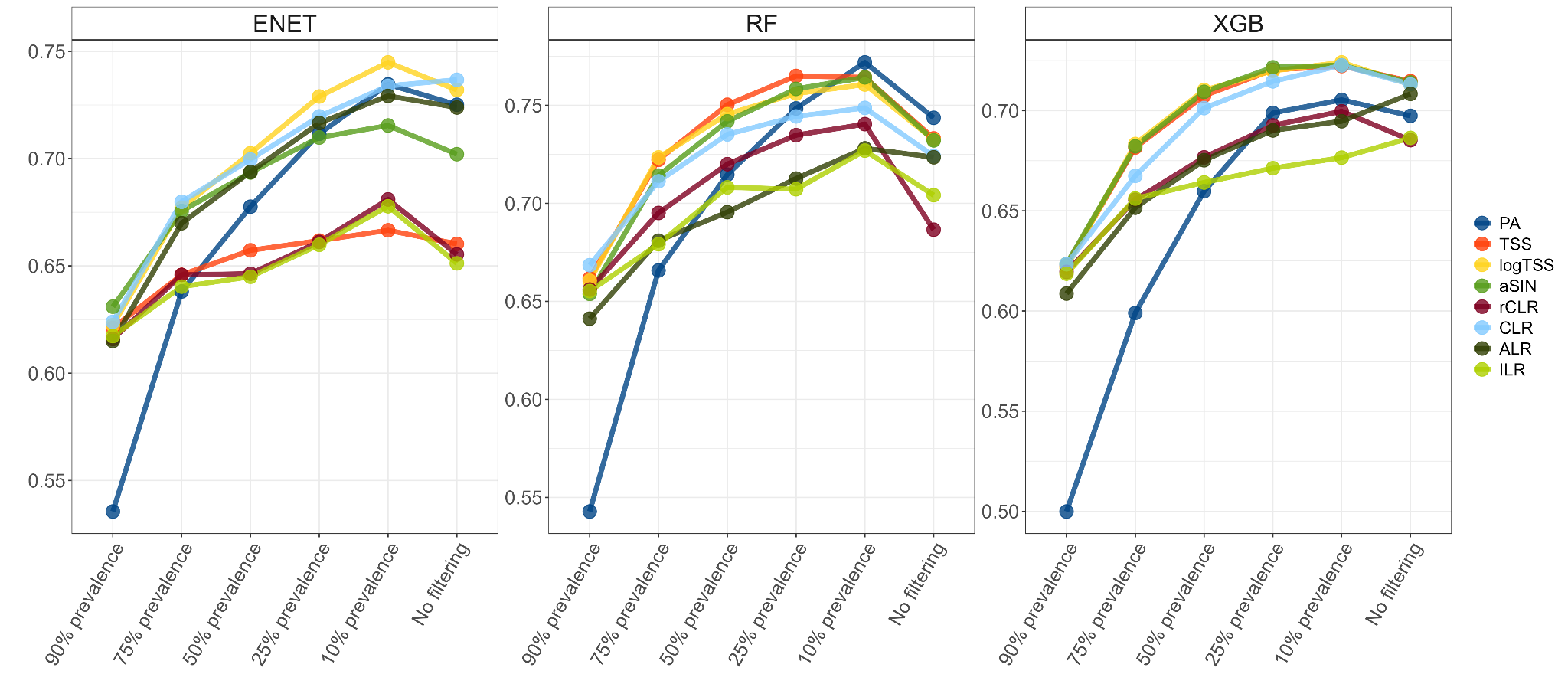


**Supplementary Figure 4**. Classifier performance dependence on the data transformation and data dimensionality (feature filtering based on the prevalence of the taxa). Colors represent data transformation.

**
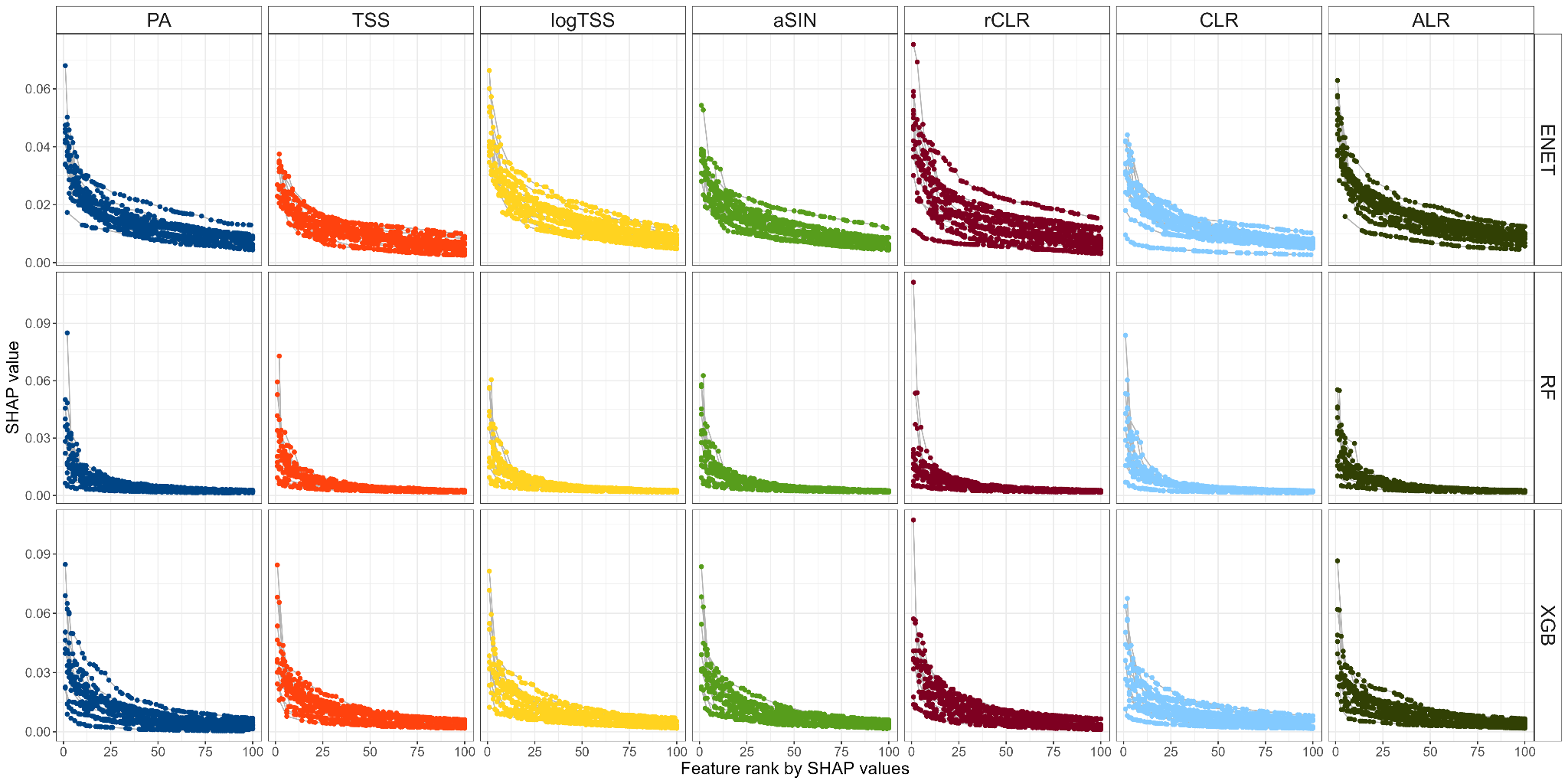
**

**Supplementary Figure 5.** Mean absolute SHAP values distribution for top 50 features across all datasets plotted separately for each transformation.


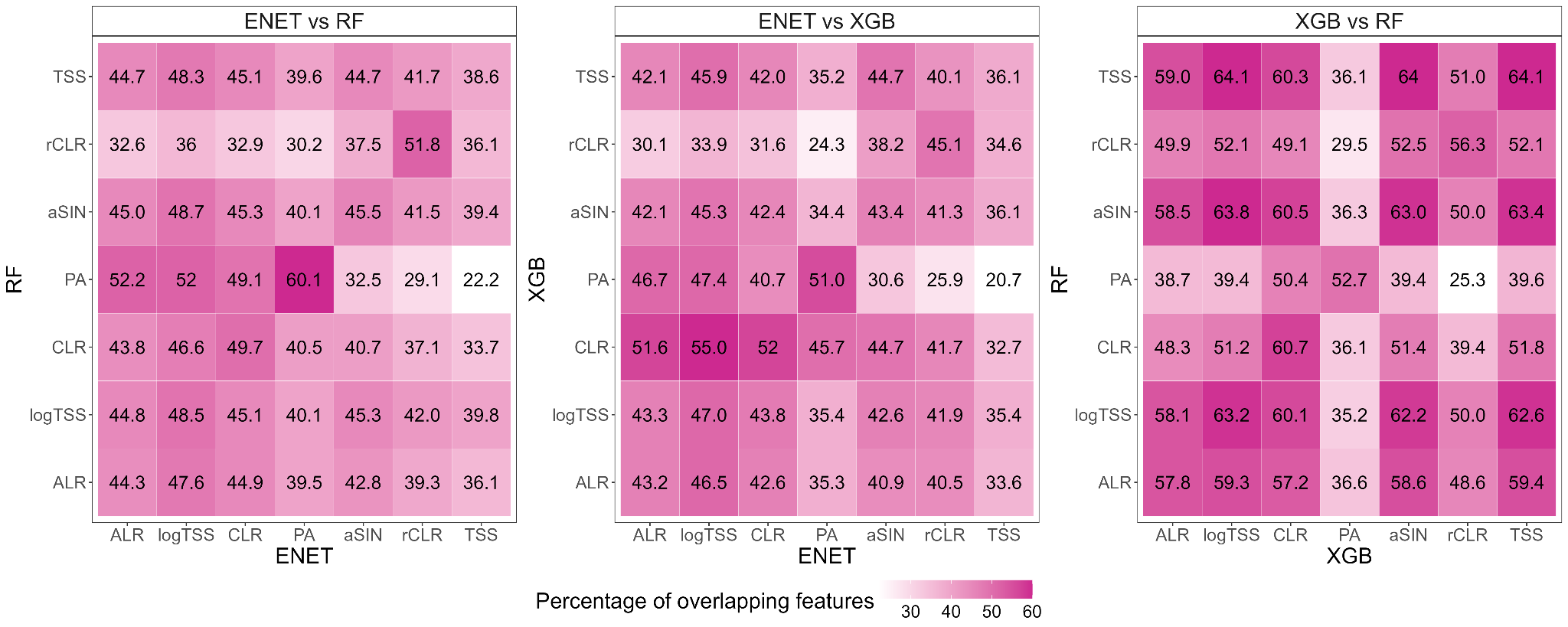


**Supplementary Figure 6.** Overlap in the most significant predictors (top 25 predictors by SHAP values) between the algorithm-data transformation combinations.


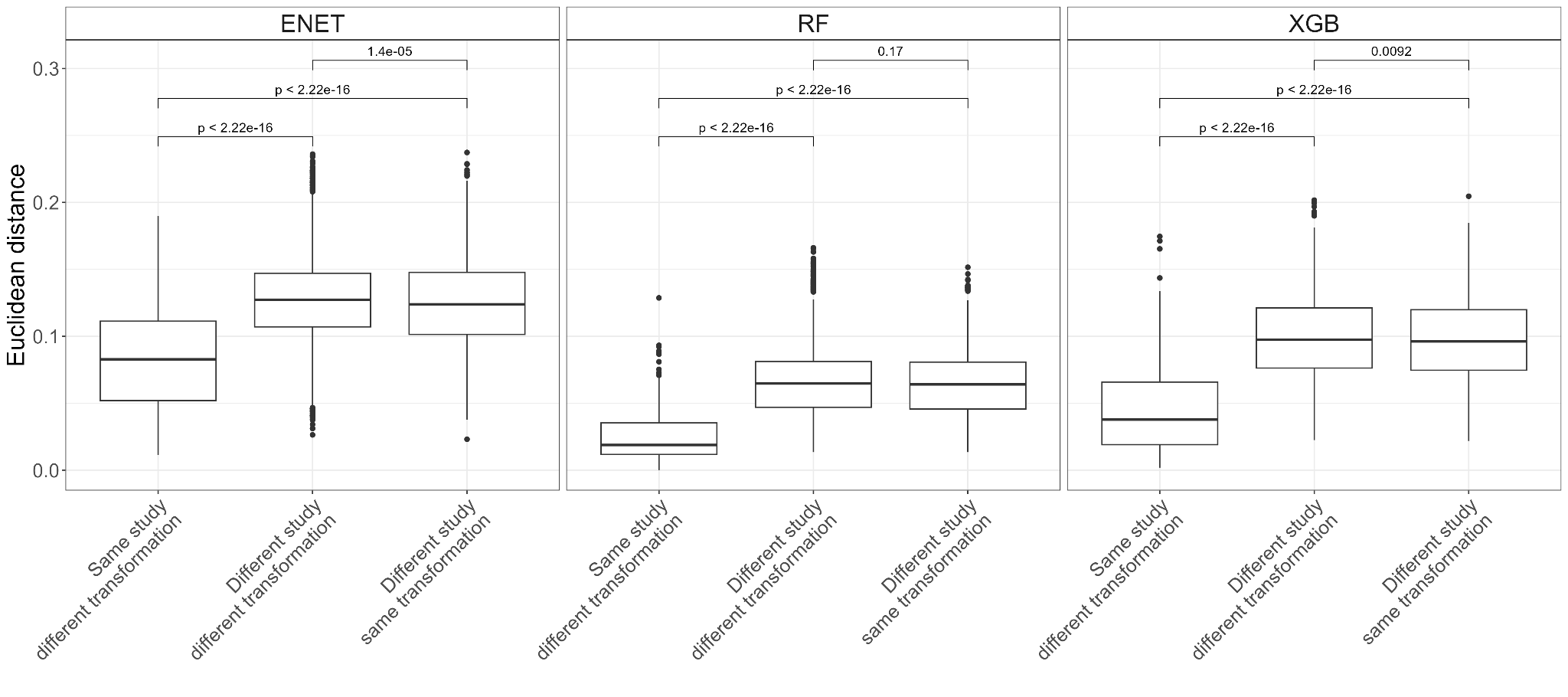


**Supplementary Figure 7**. Differences in the feature importance profiles between and within different studies and data transformations measured by Euclidean distance. P-values correspond to the unpaired t-test.

*
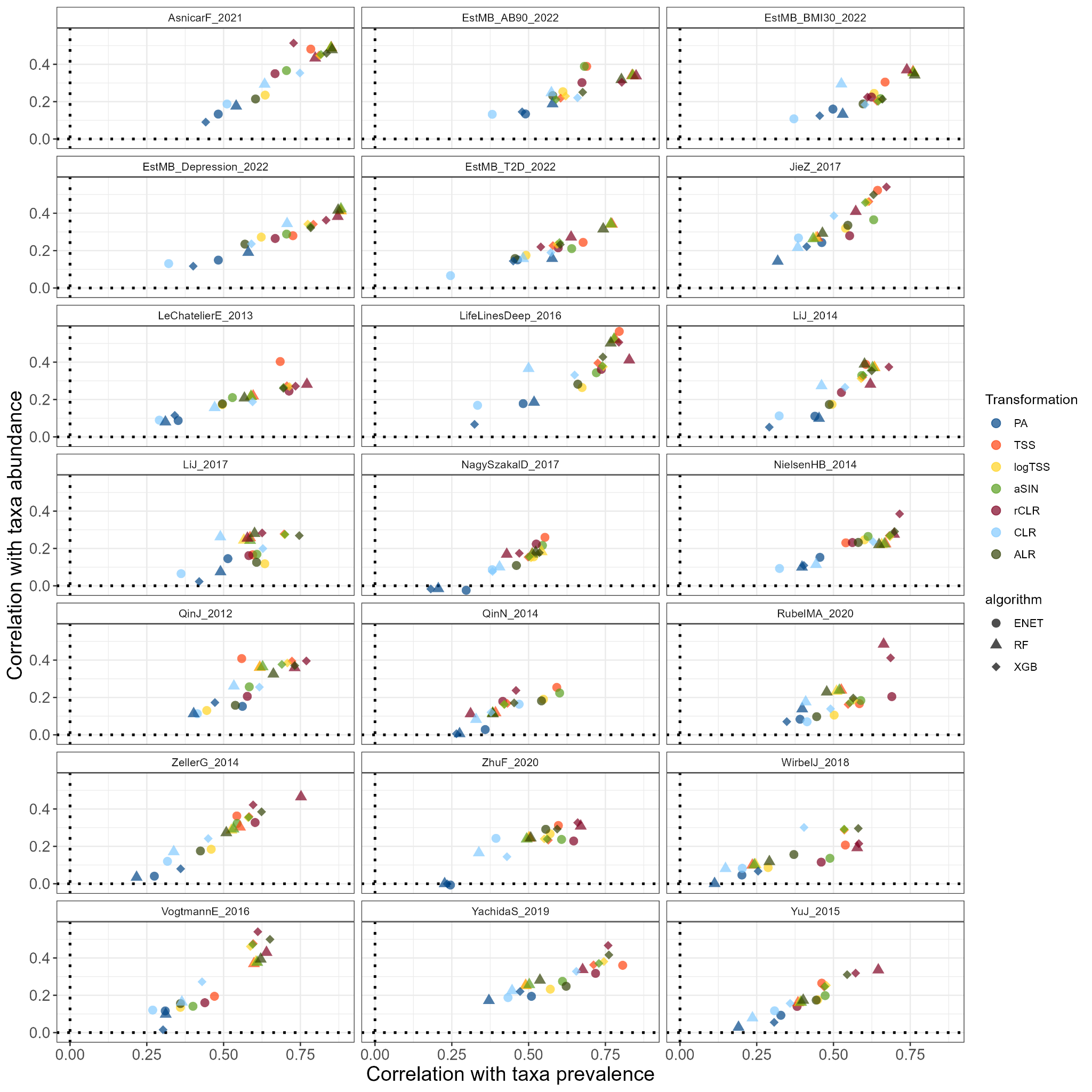
*

**Supplementary Figure 8**. Correlation between the mean absolute SHAP values and taxa prevalence/mean relative abundance. Abbreviations: *ACD - atherosclerotic cardiovascular disease; BMI - body mass index; CRC - colorectal cancer; IBD - inflammatory bowel disease; STH - soil-transmitted helminths*; *T2D - type 2 diabetes*

*
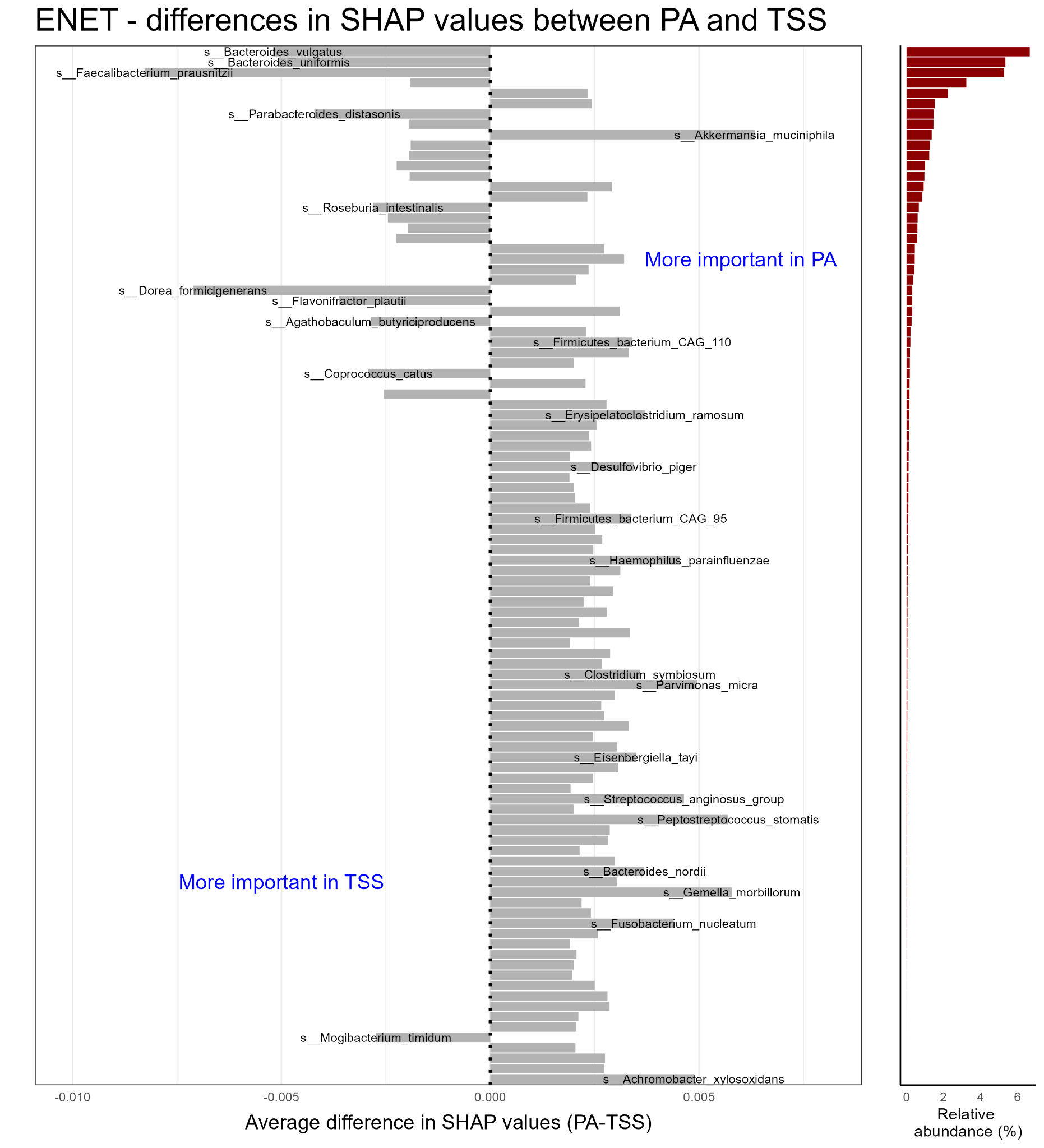
*

**Supplementary Figure 9.** Mean differences in SHAP values across all datasets between PA and TSS for elastic net logistic regression (ENET). Top 100 taxa according to the absolute mean difference in SHAP values are shown. Taxa with the largest absolute mean difference in SHAP values between PA and TSS are further highlighted (z-score >= 3). Red barplots indicate the relative abundance of these taxa in percentages.


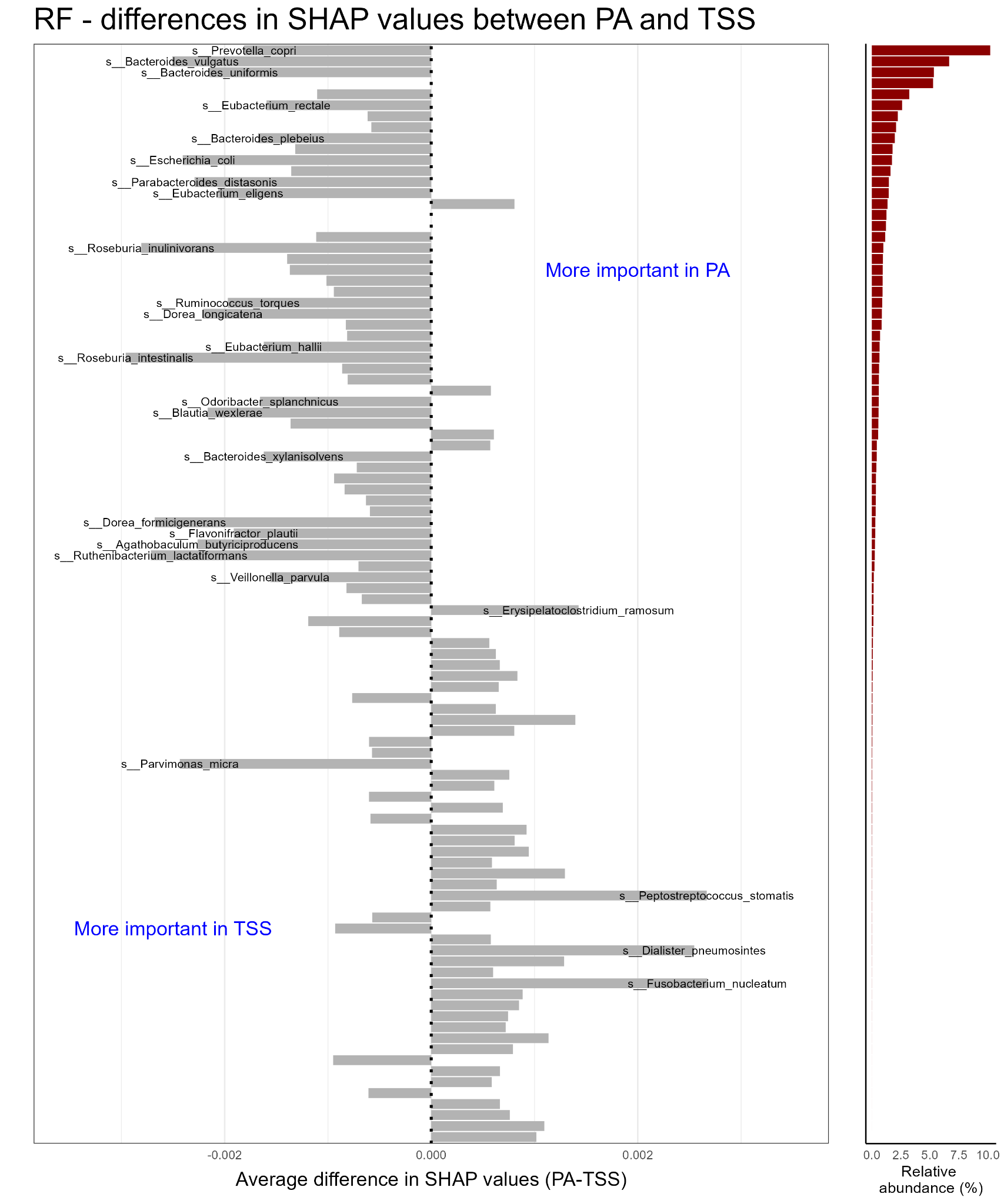


**Supplementary Figure 10.** Mean differences in SHAP values across all datasets between PA and TSS for random forest (RF). Top 100 taxa according to the absolute mean difference in SHAP values are shown. Taxa with the largest absolute mean difference in SHAP values between PA and TSS are further highlighted (z-score >= 3). Red barplots indicate the relative abundance of these taxa in percentages.


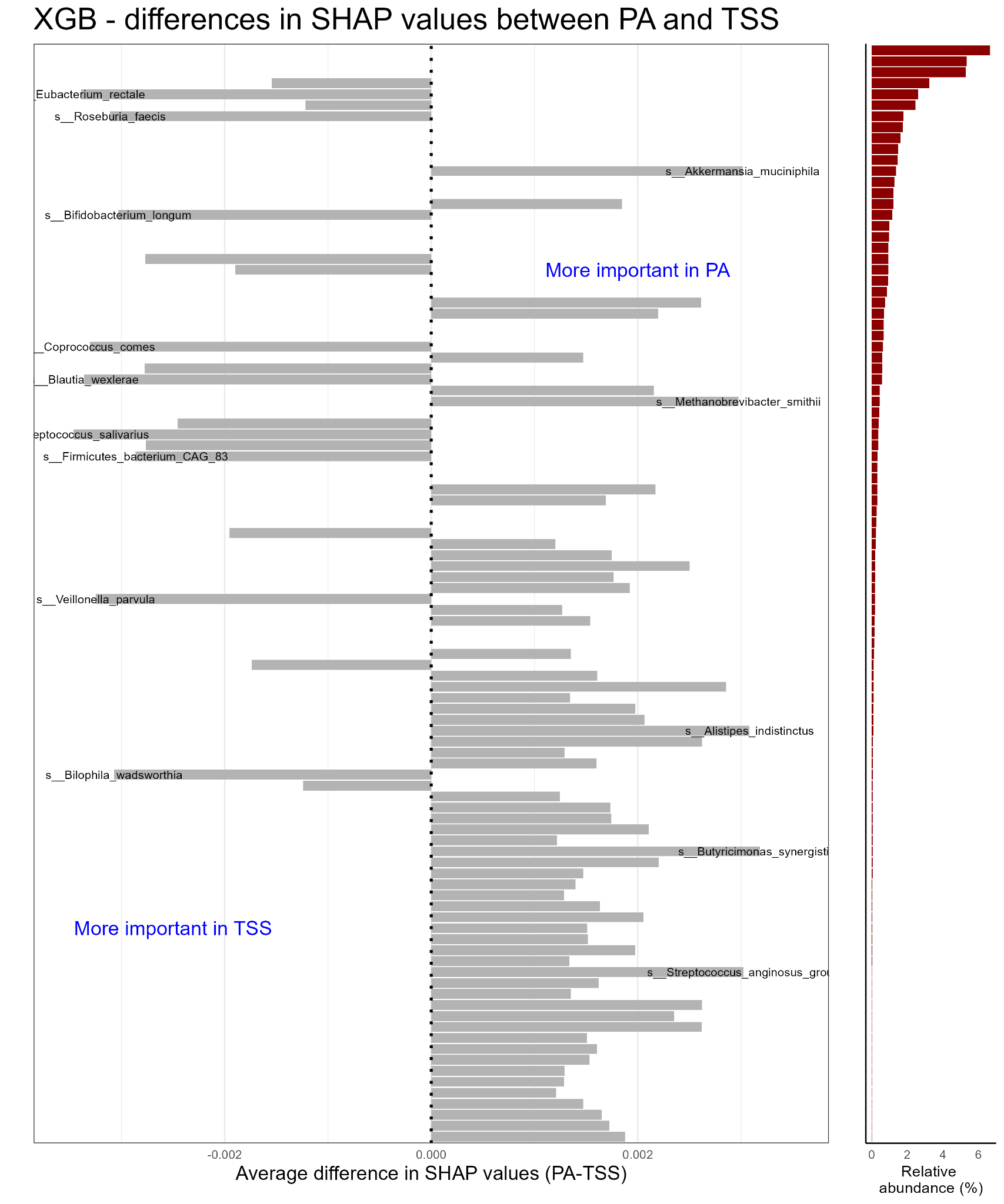


**Supplementary Figure 11.** Mean differences in SHAP values across all datasets between PA and TSS for XGBoost. Top 100 taxa according to the absolute mean difference in SHAP values are shown. Taxa with the largest absolute mean difference in SHAP values between PA and TSS are further highlighted (z-score >= 3). Red barplots indicate the relative abundance of these taxa in percentages.


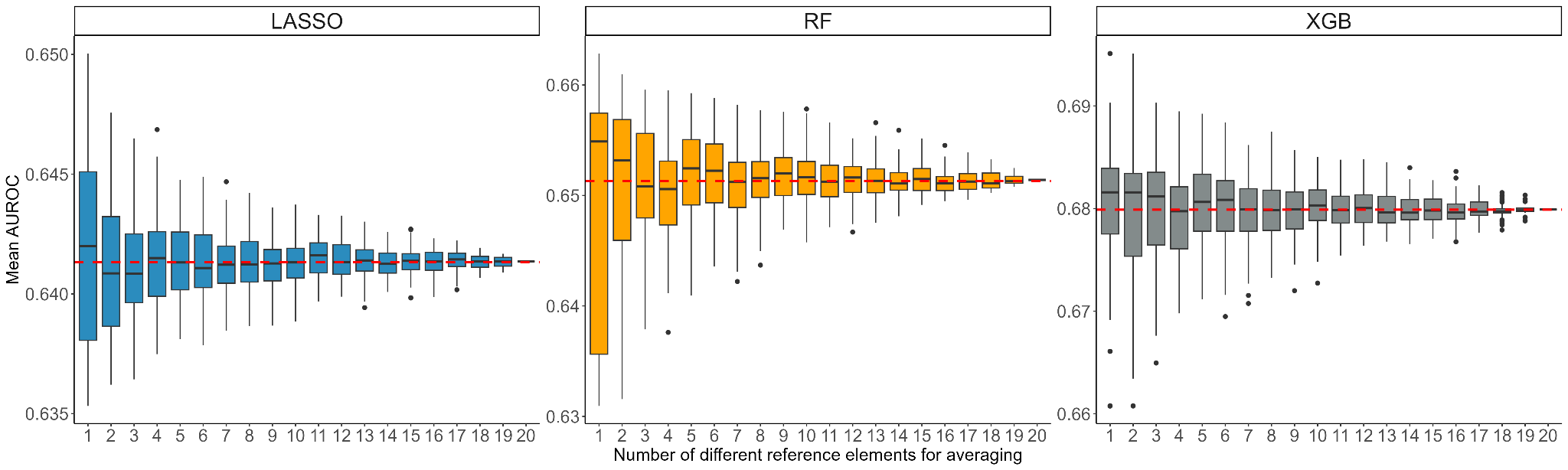


**Supplementary Figure 12**. Distribution of classifier performance for antibiotics consumption in the Estonian Microbiome cohort in respect to the number of different reference elements used for averaging for the additive log-ratio (ALR) transformation.
